# Sex differences in innate immune cell responses: Impact on cancer immunity and response to therapy

**DOI:** 10.3389/fimmu.2026.1864861

**Published:** 2026-08-03

**Authors:** Sina Ramin, Terry R. Medler

**Affiliations:** Earle A. Chiles Research Institute, a Division of Providence Cancer Institute, Providence Portland Medical Center, Portland, OR, United States

**Keywords:** cancer immunology and immunotherapy, hormones, innate immunity, myeloid cells, sex

## Abstract

Sex hormones regulate innate and adaptive immune responses to infection, autoimmunity, and carcinogenesis through genomic and non-genomic mechanisms. Estrogen, progesterone, and androgen receptors are widely expressed on immune cells and modulate their functional responses to pathological insults. While the impact of biological sex on infection, autoimmunity, and cancer risk has long been appreciated, their impact on response to cancer therapy is only beginning to be explored. Combinatorial strategies are being leveraged in the clinic to elicit antitumor immune responses, but the role of sex and hormones remains underexplored. Given that hormones impact both innate and adaptive immune responses, their interplay within the overall treatment regimen needs to be better understood, particularly for younger patients whose hormone levels remain relatively high, and for aging patients whose immune system divergence evolves despite reduced hormone levels. Herein, we review the interaction between hormones and the innate immune system and its implications for anti-cancer therapy.

## Introduction – sex, hormones, and cancer risk

**Figure 1:**
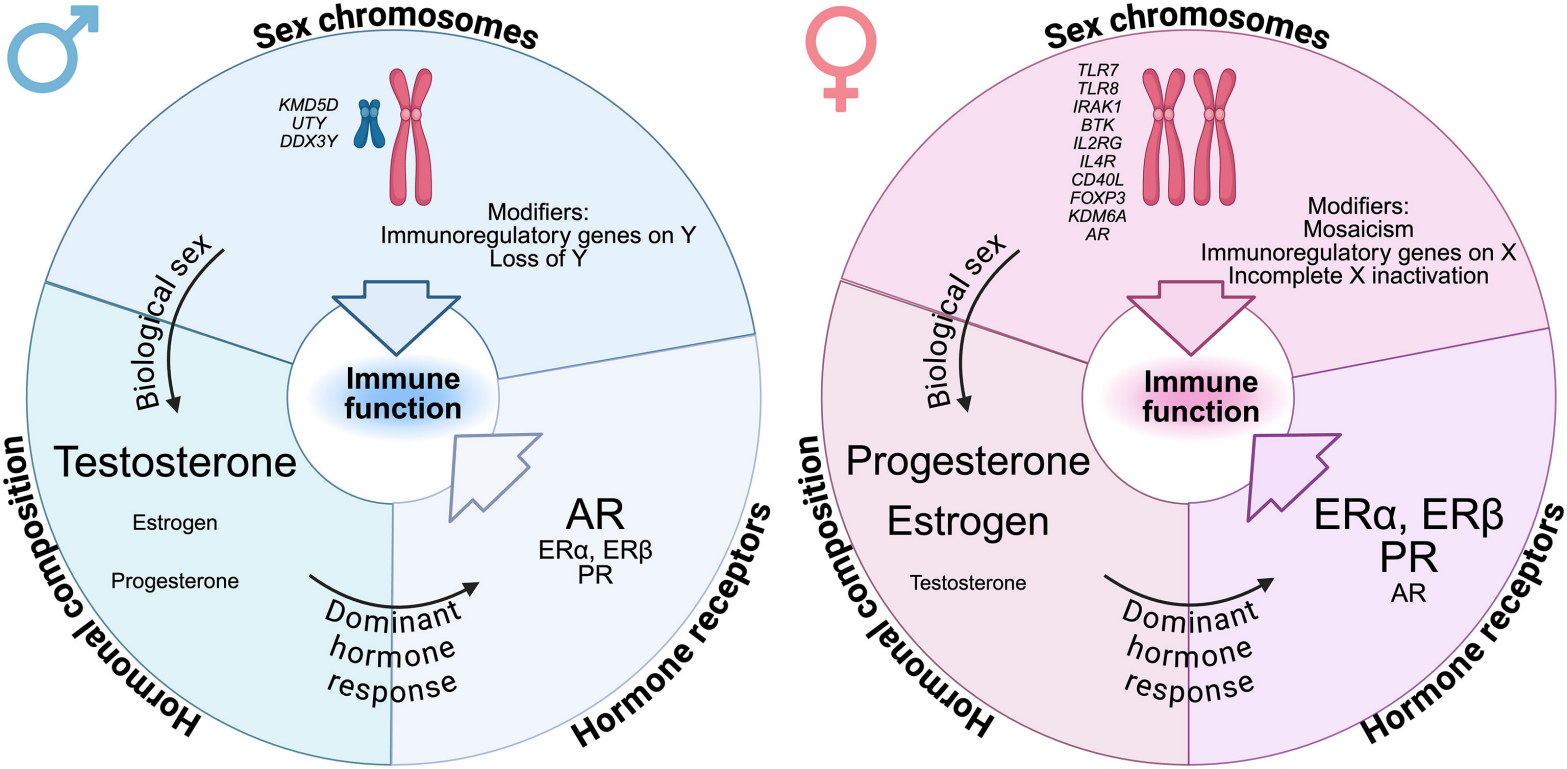
Influence of sex chromosomes, hormones, and hormone receptors on immune function. Inherited chromosomes influence biological sex and the levels of circulating hormones via sex-determinant genes on the X and Y chromosomes. Also encoded in X and Y chromosomes are immunoregulatory genes that influence immune responses. Other modifying characteristics include mosaicism and incomplete inactivation of the X chromosome, and loss of the Y chromosome in immune cells that occurs with more frequency during aging. Testosterone is the dominant hormone in males that exerts its effects through the AR expressed on immune cells, with minor contributions of estrogen and progesterone. Conversely, estrogen and progesterone are the dominant hormones in females that exert their effects through ERα, ERβ, and PR, with minor contributions from testosterone. Created with BioRender.com.

Biological sex has a profound impact on immunity and influences response to infection, autoimmunity, and cancer risk. Epidemiological studies reveal that while females are better poised to fight many bacterial and viral infections compared to males, they also have a higher propensity for autoimmune diseases (1–3). Males, on the other hand, have increased morbidity and mortality to infection compared to females (2). The difference in immunity extends to vaccine response and cancer risk. Females generally respond better to vaccines, but also have an increased risk of adverse events (4). Males are more likely to develop cancer and have higher mortality rates than females, though this trend has been narrowing in recent years (5). Among cancers shared across sexes, males have higher rates of esophageal, head and neck, gastric, bladder, lung, liver, kidney, pancreatic, colorectal, and skin cancers, while females have higher rates of thyroid and gallbladder cancers (6). Historically, the increased risk of cancer in males has been partially attributed to higher exposure to carcinogens (e.g. smoking and/or occupational exposure), but we now understand that the interplay between genetics, circulating hormones, and hormone receptor expression impact immunity and results in distinct risks to infectious diseases and cancer, and also affects responses to therapies (6–9) (**Figure 1**).

The basis of sex differences between males and females lies in the X and Y chromosomes, not just because of the sex-determinant genes encoded in them, but also because of important immune-associated genes and regulatory elements encoded in each chromosome. Females carry 2 copies of the X chromosome, and one copy becomes inactivated in each cell. This results in mosaicism throughout the host and generally protects females from diseases caused by mutations on the X chromosome, while males remain more vulnerable to X-linked hereditary defects. However, not all genes become inactivated, and it appears that a portion of tumor suppressor genes escape inactivation in females and the resulting increased expression may at least partially account for protection against cancer (10). Additionally, there are dozens of genes that are important for immunity that are expressed on the X chromosome and likely account for some immune differences between males and females due to incomplete inactivation and at least partially contribute to the increased risk of autoimmunity (11). For example, *TLR7*, *TLR8*, and *IRAK1* are expressed on the X chromosome and are important for sensing single stranded ribonucleic acid (ssRNA) from viruses and the resultant type I interferon (IFN) production (11). *TLR7* appears to escape inactivation quite readily in B cells and myeloid cells, resulting in increased transcription and responsiveness to toll-like receptor (TLR)7 ligands and likely contributes to increased risk of systemic lupus erythematosus (SLE) (12). Along those lines, *DDX3X* is also found on the X chromosome and is a critical positive regulator of type I IFN that is critical for antiviral responses (13). Several genes associated with B and T cell responses are also encoded on the X chromosome, including *BTK*, *IL2RG*, *IL4R*, *CD40L*, *FOXP3* and others (11). The X chromosome also contains *KDM6A*, an epigenetic modulator of immunity that regulates expression of major histocompatibility complex (MHC) genes (14), and regulates susceptibility to autoimmunity (15) and therapeutic responses in bladder cancer (16).

While the Y chromosome is significantly smaller and has fewer genes, it also plays an important role in shaping immune responses. In mice, genetic variation of the Y chromosome influences influenza A pathogenesis in male mice by regulating the frequency of interleukin (IL)-17^+^ γδ T cells (17), while copy number variations in regulatory genes on the Y chromosome impacts susceptibility to experimental allergic encephalomyelitis and myocarditis (18). The histone demethylase KDM5D is expressed on the Y chromosome and regulates immune evasion in colorectal cancer in part through modulation of MHC-I expression (19). Perhaps one of the more interesting aspects of the Y chromosome is that the loss of the Y chromosome (LOY) occurs relatively frequently in immune cells as males age and is associated with an increased risk of hematological malignancies, solid cancers, Alzheimer’s disease, and cardiovascular disease (20–23). Interestingly, LOY in natural killer (NK) cells was associated with Alzheimer’s disease, while LOY in CD4^+^ T cells and granulocytes was associated with prostate cancer in one study (24), indicating that cell type-specific LOY may result in certain vulnerabilities. Additionally, it was shown that LOY in immune cells correlates with LOY in tumor cells and impacts outcome across multiple tumor types (25). Mouse models are beginning to help understand how LOY impacts immunity. In the background of transgenic expression of AML1-ETO and p53 loss, immune cell LOY increased acute myeloid leukemogenesis in mice (26). In another study, aged mice with immune cell LOY had increased fibrosis and cardiac dysfunction, and LOY was associated with worsened outcomes of experimental heart failure (27). The increased fibrosis was linked to a macrophage population with a fibrotic phenotype associated with transforming growth factor (TGF)β signaling (27). Together, these studies indicate that sex chromosomes play an important role in protective immunity and have a profound impact on disease pathogenesis.

**Figure 2:**
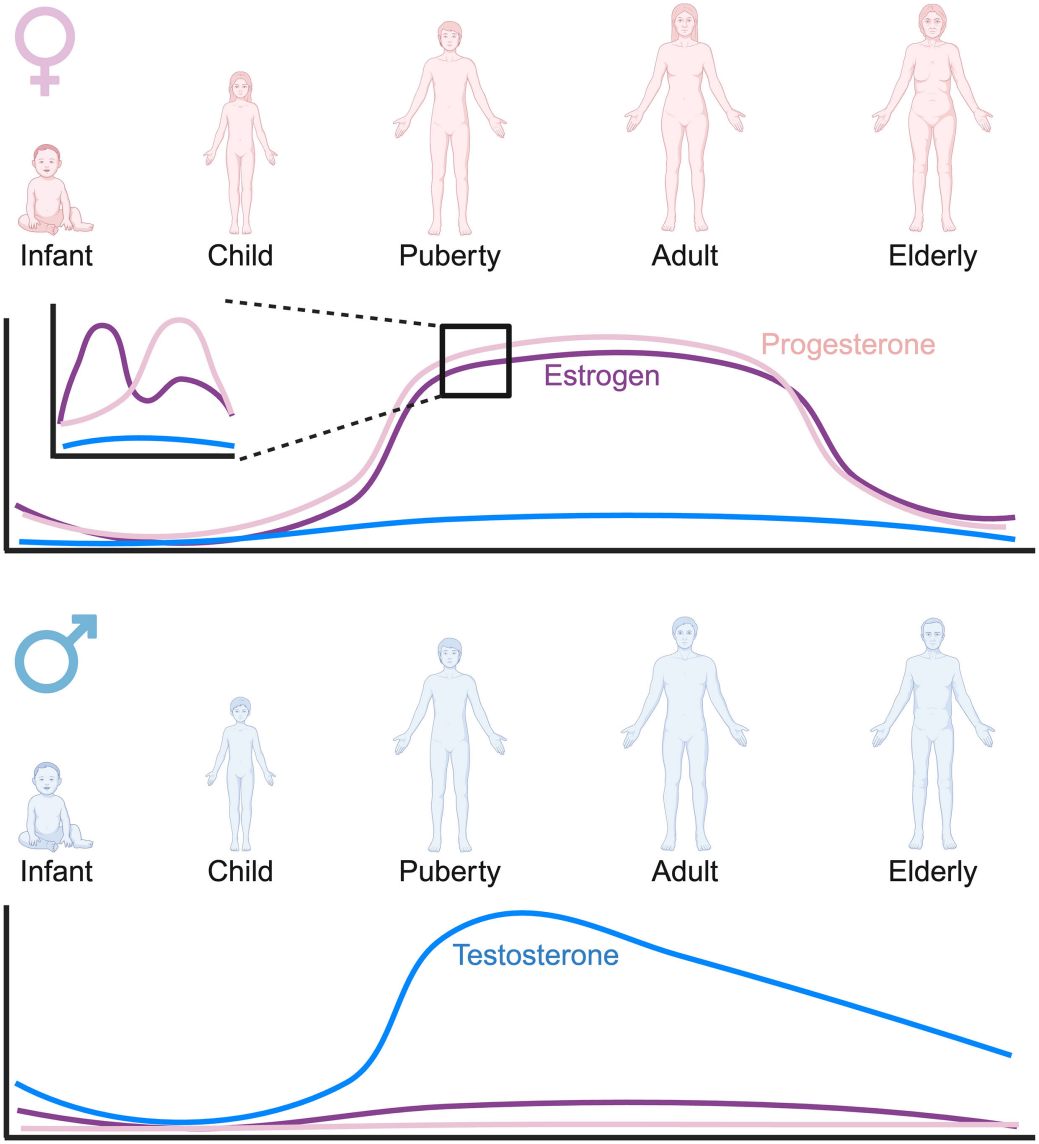
Relative levels of circulating hormones in females and males. Infants and children have relatively low levels of sex hormones. Upon reaching puberty, levels of estrogen and progesterone spike in females (top) and reach peak levels in mid-adulthood, with the relative maximum hormone levels depicted. During the menstrual cycle (inset), estrogen levels peak during the follicular phase and then quickly drop during ovulation while progesterone levels are quickly rising. Progesterone levels peak mid-luteal phase and then drop to baseline during menstruation. In males, testosterone levels sharply rise during puberty and reach their maximum levels during early adulthood. Testosterone levels then steadily drop throughout their lifetime. Created with BioRender.com.

Along with the differences in sex chromosomes are circulating hormones that differ between males and females across their lifespan (**Figure 2**); males have higher levels of circulating testosterone and dihydrotestosterone, while females have higher levels of estrogen and progesterone. These hormones are well described to promote sex-specific cancers due to epithelial expression of hormone receptors (e.g. estrogen and progesterone increase breast and endometrial cancer risk for females, while testosterone increases prostate cancer risk in males) (28, 29), but virtually all immune cells also express hormone receptors and the resultant immunity is shaped by the relative hormone concentrations and receptor expression (**Box 1**). In females, cycling hormone levels can be observed during menstruation and pregnancy that impact immune responses. Female immune responses tend to skew towards T helper (Th)1 responses and is reinforced by increasing levels of estrogen during the follicular phase. After ovulation, progesterone levels increase and peak mid-luteal phase resulting in immunosuppression and skewing towards Th2 responses until menstruation, when hormone levels drop and again skew towards Th1 responses. These hormonal changes lead to a window of vulnerability of sexually transmitted infections (STIs) during the relatively immunosuppressive environment of the luteal phase (30–33). Similarly, the increased levels of progesterone during pregnancy promote a Th2 environment, resulting in increased risk of certain infections (34, 35). Many females also take oral contraceptives that contain progestins – synthetic progesterone-like compounds that exert their effects by binding to the progesterone receptor (PR). Progestins have also been implicated in promoting immunosuppression, resulting in increased risk of STIs, and altering outcomes for certain gastrointestinal and respiratory tract infections (36). Conversely, males are generally more susceptible to infections and have worse outcomes in part due to the immunosuppressive effects of androgens (2, 37). These immunosuppressive effects likely extend to cancer as well. In a rat model of carcinogen-induced precancerous pancreatic lesions, decreased testosterone levels associated with orchiectomies in male mice resulted in reduced number of pancreatic lesions, while ovariectomized females had an increase in the number of lesions, and hormone replacement with either estrogen or testosterone largely reverted the respective phenotypes (38). Similar results were shown for a chemical-induced model of renal cell carcinoma in rats (39). While these studies did not examine whether the effects were due to epithelial or immune-mediated effects, it remains plausible that there is at least a partial contribution from the immune compartment.

Box 1 **Hormone signaling.**

Canonical hormone signaling occurs when the hormone diffuses into a responsive cell and binds to its receptor in the cytoplasm or nucleus, resulting in conformational changes, dimerization, and nuclear localization of the receptor:hormone complex. The complex and recruited cofactors bind to hormone response elements within promoters to drive gene transcription. Non-canonical signaling can include membrane localized receptors, or alternatively hormone binding G protein-coupled receptors. The palmitoylated membrane-localized hormone receptors facilitate rapid activation or repression of signaling cascades from growth factors, non-growth factor tyrosine kinases, or G proteins (237).

*Estrogens and estrogen receptors:* Bioactive estrogens include estrone (E1), 17β-estradiol (E2), estriol (E3), estetrol (E4), and 27-hydroxycholesterol. Of these, E2 is the most abundant and has the highest affinity for all estrogen receptors. Here, estrogen refers to E2 unless otherwise specified. Estrogen receptor (ER)α and ERβ are the classic estrogen receptors that drive canonical estrogen signaling. They are encoded by the *ESR1* and *ESR2* genes, respectively, and have variable expression patterns within different tissues. ERα is the most widely expressed in immune cells, though ERβ is also likely expressed in some immune cells but remains unclear due to questions regarding reagent specificity (238). The third receptor that binds estrogen is G protein-coupled estrogen receptor 1 (GPER), a G protein coupled receptor (GPCR) primarily localized to the endoplasmic reticulum and Golgi apparatus (239). Estrogen binding results in activation of adenylyl cyclase and production of cAMP, Rho-ROCK/YAP-TAZ signaling, and activation of PI3K/AKT via potentiation of epidermal growth factor signaling. GPER is expressed on many immune cells, including lymphocytes, neutrophils, eosinophils, monocytes, and macrophages (240).

*Progesterone:* Progesterone and the synthetic progestins commonly used for birth control and hormone replacement therapy primarily function through the progesterone receptor (PR). PR is also widely expressed in most immune cells, including macrophages, DCs, neutrophils, mast cells, NK cells, and B and T cells. PR is transcribed from the *PGR* gene and has 2 main isoforms, PR-A and PR-B (241). PR-B is the full-length receptor, while PR-A lacks the first 164 amino acids of the N-terminal domain that contains transcription activating domain 3 (AF3) (242). PR-A and PR-B have overlapping and non-overlapping functions. PR-B is generally considered a stronger activator of transcription due to the ability of AF3 to synergize with other activating domains within the receptor (243). PR-A is transcriptionally active (244), but also represses PR-B-mediated transcription (245). In a model where relative levels of PR-A and PR-B could be tuned, increased PR-A expression was associated with increased proinflammatory gene transcription (244). PR-C is another isoform transcribed from the PGR locus that lacks the first 594 amino acids inclusive of the N-terminal domain and a portion of the DNA binding domain. While PR-C lacks DNA binding capabilities, it can homodimerize to sequester progesterone or heterodimerize with PR-A or PR-B to modulate receptor activity. There are several other isoforms of PR reported, including PR-M, PR-S, PR-T, and several splice variants lacking full or partial exons, but their effects have not been fully elucidated (246–249). Other progesterone receptors include membrane progesterone receptors (mPRs) transcribed by the progesterone and adiponectin Q receptor (PAQR) gene family that include mPRα (*PAQR7*), mPRβ (*PAQR8*), and mPRγ (*PAQR5*). These mPRs recruit inhibitory or activating G proteins that regulate cAMP production and PKA activation, intracellular calcium release, and activate MAPK/ERK and PI3K/AKT signaling cascades. Progesterone receptor membrane components 1 (PGRMC1) and 2 (PGRMC2) are single membrane-spanning receptors located on the plasma membrane and organelles that bind heme and progesterone (250). They regulate calcium release and activation of protein kinase G and may play a role in cell cycle regulation and survival (251).

*Testosterone:* Testosterone and 5α-dihydrotestosterone (DHT) are the primary circulating androgen receptor (AR) agonists. Testosterone can be converted to more highly active DHT by 5α reductase and to E2 by aromatase. The AR is expressed in most immune cells, including macrophages, DCs, neutrophils, eosinophils, mast cells, NK cells, and B and T cells (52). The AR is located on the X chromosome and exists in two isoforms much like PR, where AR-B is the full-length receptor and AR-A lacks the first 187 amino acids (252, 253). The relative levels differ among tissues, but AR-A represents 4-26% of the total AR protein within samples (253). AR-A appears to be less transcriptionally active than AR-B, likely through the reduced ability to bind coactivators by the truncated N-terminal domain (254). Dozens of splice variants are described in castration-resistant prostate cancer patients, likely due to the selective pressure of androgen deprivation therapy (255–259). Their transcriptional activity replicates many actions of the AR, but isoform-specific regulation has also been described (260). While these splice variants commonly arise in advanced prostate cancer, they have not been described in immune cells. Testosterone can also signal via G protein-coupled receptor class C group 6 member A (GPRC6A), a multiligand G protein-coupled receptor (261–263). While DHT is a more potent agonist of AR compared to testosterone, it does not appear to bind GPRC6A at physiological levels (262, 264). GPRC6A agonism regulates activation of adenylyl cyclase and cAMP production, activation of MAPK/ERK pathway, intracellular calcium release and activation of PI3K/AKT/mTORC1 pathway. There are other candidates for transmembrane receptors that bind androgens, including the androgen-regulated Ca^2+^ cation channel TRPM8 (265, 266), the zinc transporter ZIP9 (267–269), and the GPCR OXER1 (270), but their effects are less studied.

While the causes of dichotomous risk based on sex are multifactorial, the resulting phenomenon may also be partially explained by differences in immune composition between the sexes. Higher levels of circulating B cells, mucosa-associated invariant T (MAIT) cells, neutrophils, and increased CD4:CD8 T cell ratios are observed in females (40–43), while there are increased monocyte, myeloid cell, NK cell, and Treg populations in males (44–47). These differences in composition correspond to increased T cell activity in females compared to males (48). In a survey of 91 healthy donors, RNA sequencing (RNAseq) of various populations of monocytes, NK cells, B cells, and T cells revealed 1875 unique transcripts that were differentially expressed between sexes and remarkably, 72% of the transcripts were uniquely expressed within a single immune cell type in a sex-dependent manner (49). Upstream analysis indicated enrichment of TLR signaling and type I and type II IFN signaling in female subjects (49). Another analysis found increased gene set enrichment analysis (GSEA) scores for IFN response, complement, IL6/JAK/STAT, and coagulation across immune populations isolated from the spleen and peritoneal cavity of females (50). These transcriptional differences, alongside the increased CD4:CD8 T cell ratio and other cellular differences may at least partially explain why females tend to have stronger B and T cell responses compared to males.

**Figure 3:**
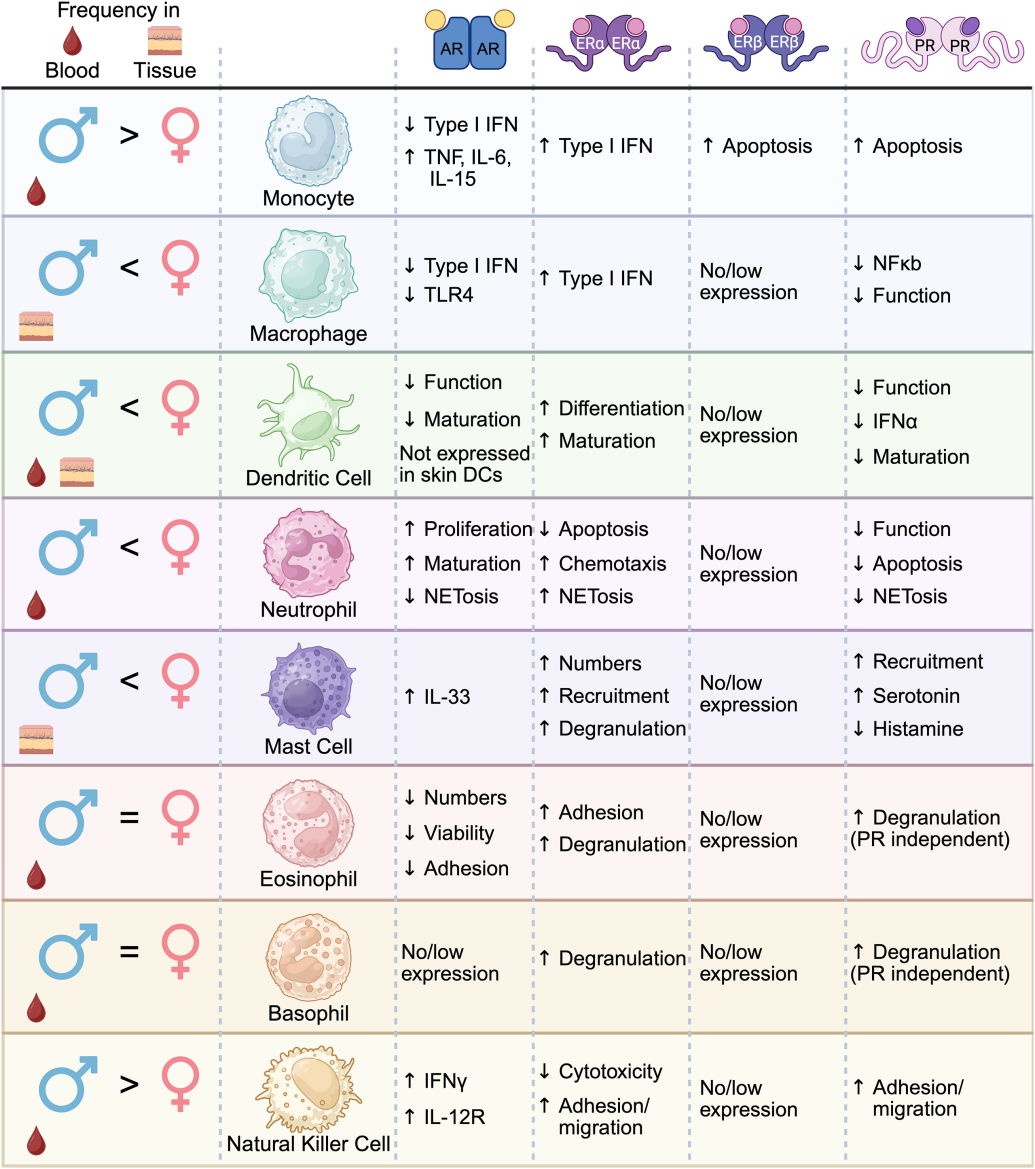
Hormonal effects on innate immune cells. Relative quantity of immune cells in males vs females in blood and/or tissue (left). Summarized effects of hormone:receptor interactions on immune function (right). Created with BioRender.com.

Together, these studies indicate that inherited sex chromosomes are the underpinnings of these sex-biased immune effects, not only due to the sex-determinant genes that result in different levels of circulating gonadal hormones, but also due to regulatory genes encoded in the X and Y chromosomes. Immune regulatory genes encoded in sex chromosomes result in differing abilities to sense pathogens and damaged cells, thereby shaping the magnitude of response to invading pathogens. Hormone receptors expressed on immune cells responding to circulating hormones that fluctuate throughout the life of the individual further shape the immune response and add an additional layer of regulation to immune cell gene transcription profiles. The final layer of complexity is shaped by tissue-specific cues, interactions with the microbiome, tumor genetics, and therapeutic context. It has become increasingly evident that interactions between sex, hormones, aging, and cancer affect therapeutic responses and recent reviews have covered various aspects of sex bias and the role of hormones in cancer (51–53). Myeloid cells are critical regulators of the overall immune response and are influenced by these interacting layers of regulation (**Figure 3**). Because myeloid targeting therapies have entered the clinic, it is imperative that we understand how impacted cells are influenced by sex, hormones, and aging to design rational combinatorial strategies targeting the populations most likely to benefit. Below we summarize known effects of sex and hormones on individual myeloid cell types in infection, inflammation, cancer, and response to therapy.

## Monocytes and macrophages

Monocytes and macrophages have been shown to be different not just by number, but by function between males and females. Males tend to have higher levels of circulating monocytes (47), and it was shown that administration of testosterone increased monocyte counts in two randomized trials of otherwise healthy men (54). Conversely, estrogen suppresses circulating monocyte numbers; premenopausal females have lower numbers of monocytes compared to menopausal females, and hormone replacement therapy results in a decrease of monocytes comparable to levels observed in premenopausal females (55). As a potential mechanism of the impact of estrogen on monocyte numbers, ERβ has been shown to regulate FasL expression in monocytes, resulting in increased apoptosis and reduced numbers of circulating monocytes (56). Progesterone also appears to influence monocyte numbers by decreasing levels of the survival factor Bcl-2, resulting in increased apoptosis (57). Despite the effects of estrogen and progesterone on the number of circulating monocytes, females generally have higher numbers of tissue resident macrophages, particularly in the peritoneum and pleural space (58). However, it appears that males accumulate an increased number of macrophages in adipose tissue that are more inflammatory when fed a high fat diet, indicating that there may be some tissue-specific differences in numbers between males and females under certain pathological conditions (59). The increased macrophages in adipose tissue of male mice were due at least in part to increased leptin receptor and free fatty acid receptor GPR120 expression on macrophages from male mice that resulted in increased migration in response to the increased leptin and free fatty acids (59).

Additional studies using RNA and ATAC sequencing have sought to delve into sex specific differences between immune populations. RNA and ATAC sequencing of major immune populations from the spleen and peritoneal cavity revealed few notable differences between males and females in most unstimulated immune cells, with the exception of macrophages (50). Pathway analysis revealed gene set enrichment signatures in female macrophages involved in IFN response, complement, IL6/JAK/STAT, and coagulation pathways (50). Perhaps most interestingly, the tissue of origin was the primary driver of sex-specific gene expression differences among macrophages from different tissues (50).

As mentioned above, females tend to express higher levels of type I IFN at baseline compared to males. There are a number of mechanisms by which Type I IFN is produced. TLRs expressed on immune cells recognize pathogen-associated molecular patters (PAMPs) common amongst pathogens and danger-associated molecular patterns (DAMPs) that are released from stressed or dying cells. This leads to signal transduction through the TLR/MyD88/NFκB that drives inflammatory cytokine production and/or the TLR/(MyD88/TRAF/TRAM/TRIF)/IRF pathway to drive Type I IFN production [reviewed in (60)]. Due to incomplete X inactivation, TLR7 and TLR8 are more highly expressed on female macrophages and likely accounts for increased IFN production. In support of this, when macrophages from male and female mice were stimulated with IFNα, the genes induced were identical, but the level of induction was significantly higher in female macrophages (50). In transgender men receiving gender-affirming hormone therapy it was revealed that monocyte responses change over time. Stimulation with a TLR7/8 agonist resulted in attenuated type I IFN responses, while stimulation with TLR4 agonist potentiated responses resulting in enhanced production of TNF family proteins, IL-6, and IL-15, thereby underscoring a direct effect of hormones on effector responses (61).

Androgens have been shown to regulate expression of some TLRs on macrophages and regulate their activity. Exogenous treatment of macrophages with testosterone resulted in decreased TLR4 expression, while reducing circulating androgens in male mice by gonadectomy resulted in increased TLR4 expression and increased proinflammatory responses to endotoxin (62). As such, there is increased TLR4 expression on female macrophages that results in increased ability to phagocytose (63). However, progesterone tends to have an inhibitory effect on TLR signaling in macrophages. For example, bone marrow derived macrophages were stimulated with LPS, IL-4 or a combination to promote classical or alternative activation, which resulted in a decrease in expression of iNOS and arginase with any treatment (64). However, several other genes associated with activation were differentially regulated dependent on polarization (64). The inhibitory effect of progesterone on LPS stimulation has been attributed to increased expression of SOCS1, which negatively regulates NFκB activation and prevents downstream gene expression (65). Macrophages also express the membrane progesterone receptor mPRα and respond to extracellular progesterone by increasing expression of the proinflammatory genes *Ptgs2*, *Il1b*, and *Tnf (*66).

While these findings indicate that females tend to have increased type I IFN production associated with an M1 macrophage phenotype, the effects of hormones are also likely tissue- or stimulus-dependent. In the normal mouse mammary gland where ER is abundantly expressed, estrogen treatment was associated with increased macrophage presence and M2 macrophage polarization, while ovariectomy decreased macrophage presence and M2 macrophage polarization (67). Similarly, estrogen supplementation in ovariectomized female mice enhanced M2 polarization in a model of allergic asthma, an effect that was dependent on ERα (68). Estrogen has also been shown to promote tumor growth independent of cancer cell expression of ERs. In mouse xenografts of ER^-^ breast cancers, estrogen supplementation promoted recruitment and activation of a pro-angiogenic population of bone marrow derived macrophages that promoted tumor growth (69). Similarly, increased M2-like macrophage presence was observed in a mouse xenograft model of ERα^-^ high grade serous ovarian carcinoma upon estrogen supplementation of ovariectomized mice (70), thereby underscoring the context-dependent nature of these hormonal effects.

## Dendritic cells

As mentioned above, there is a female sex bias in response to vaccines and dendritic cells are critical for this response (4). Estrogen has been shown to influence both the granulocyte/macrophage-colony stimulating factor (GM-CSF)- and FMS-like tyrosine kinase 3 ligand (Flt3L)-mediated pathway of dendritic cell (DC) differentiation and maturation. The GM-CSF-mediated pathway is highly important for DC differentiation and maturation during inflammation and generates DCs that are more akin to dermal Langerhans cells and inflammatory/monocyte-derived DCs, while the Flt3L pathway generates a more tolerogenic DC and is more important for homeostatic DC generation and function (71). In culture, estrogen increases GM-CSF-mediated differentiation DCs from bone marrow (72). It was shown that ERα^-/-^ mice have reduced DCs differentiation compared to WT mice, due in part to decreased IRF4 expression, which is necessary for DC differentiation (73–76). DCs lacking ERα or differentiated in the absence of estrogen tend to express lower levels of MHCII and costimulatory receptors prior to activation compared to those cultured in the presence of estrogen, indicative of reduced maturation (76, 77). Conversely, DC cultured in the presence of estrogen respond to TLR ligands more effectively, with increased expression of CD86, MHCII, and increased production of IL-12 (72, 78). In contrast with GM-CSF-mediated differentiation, estrogen inhibited survival of Flt3L-mediated differentiation of DCs by signaling through the AF-1 domain of ERα (75, 79). While numbers of Flt3L-derived DCs were lower, their relative maturation appeared to be increased and produced higher levels of IL-12 when stimulated with TLR9 agonist (75). These data indicate that estrogen is an important regulator of dendritic cell differentiation and maturation during inflammatory insults.

Progesterone has been shown to inhibit DC function. When treated with progesterone, bone marrow-derived DCs had reduced secretion of the proinflammatory cytokines TNFα and IL-1β, downregulation of the activation markers MHC class II and CD80, and a reduced ability to stimulate proliferation of T cells (80). Other studies have shown that addition of progesterone decreases DC activation and release of IL-12, IL-1, and IL-6 dependent on binding to PR (81).

Testosterone does not appear to affect DC differentiation from bone marrow (72), but instead acts to alter DC function. Androgen replacement therapy in type 2 diabetic males resulted in suppressed DC function; testosterone decreased the percentage of peripheral IL-1β^+^ CD16^+^ DCs and IL-1β^+^ CD33^hi^ myeloid DCs, while the levels of IL-6 and TNFα were reduced in CD16^+^ DCs (82). In mice, reduced testosterone via orchiectomy resulted enhanced DC maturation and exhibited increased expression of MHC and costimulatory molecules (83). Similarly, when monocyte-derived dendritic cells were induced to mature with prostate cancer cell lysates, those treated with an AR antagonist expressed higher levels of the maturation markers CD86 and MHCII and reduced levels of TGFβ and IL-10 (84). Skin-resident DCs do not express the AR (85, 86), are comparatively infrequent in the skin of male mice compared to female mice, and exhibit a less activated phenotype in response to skin infection, but are nevertheless dependent on male sex hormones (87). Instead, AR-expressing type 2 innate lymphoid cells are the primary source to GM-CSF in the skin that control DC recruitment and function. Upon binding androgen, type 2 innate lymphoid cells reduce GM-CSF production, leading to reduced presence and maturation of skin DCs in male mice (87).

Plasmacytoid dendritic cells are a type of dendritic cell found in blood and lymphoid organs that specialize in detecting viral infections by TLR7 and TLR9 and respond by secreting large amounts of type I IFN. When blood samples were incubated with a synthetic TLR7 ligand, increased IFNα was observed in pDCs from females compared to males (88). These effects were in part attributed to the increased frequency of pDCs found in peripheral blood mononuclear cells (PBMCs) in females (89–91). When TLR7 was stimulated with HIV-1-derived ligands, there were also a higher percentage of IFNα^+^ pDCs derived from females that positively correlated with serum progesterone levels (91), indicating a higher degree of pDC activation upon TLR7 ligation in the presence of increasing levels of progesterone. Supporting a role for estrogen in regulating pDC function is a study where increased IFNα production was observed from blood pDCs in postmenopausal females receiving estrogen supplementation (90). Similar to bone marrow-derived DCs, progesterone and progestins reduce pDC function. Progestins reduce IFNα production in response to TLR ligands or inactivated virus (92, 93).

In males, testosterone appears to inhibit pDC function. pDCs produce less type I IFN compared to females in response TLR7/8 agonism (94) and to SARS-CoV-2 mRNA vaccines (95). As a more direct measure of the hormonal effects on pDC function, transgender men were followed over the course of a year of beginning gender affirming hormone therapy. They found reduced type I IFN production and reduced expression of IFN-regulated genes in PBMCs after beginning testosterone administration (96). An in-depth analysis of immune populations in a similar study revealed that in addition to fewer pDCs present in transgender men receiving testosterone, phenotypic differences were also observed, including increased CD81 expression and decreased type I IFN production in response to TLR7/8 ligands (61).

## Neutrophils

While females tend to have higher numbers of neutrophils compared to males, there is some evidence that androgens impact circulating neutrophil counts. In the two randomized trials described above, it was shown that administration of testosterone increased neutrophil counts in otherwise healthy men (54). Conversely, prostate cancer patients on androgen deprivation therapy have reduced numbers of mature neutrophils (97), and reversible neutropenia has also been observed as a relatively rare side effect in males with prostate cancer who are taking androgen blocking therapies (98, 99). In mice, orchiectomized and AR knockout mice have reduced neutrophils, but only neutropenia in orchiectomized mice can be reversed with androgen supplementation, indicating that AR is necessary for the observed effects (100). On the other hand, females with hyperandrogenism have neutrophilia that can be normalized with androgen blockade (101). Analysis of neutrophil precursors indicated that rather than affecting myeloid lineage commitment, androgens promote proliferation and maturation of neutrophil precursors (100). As such, neutrophils from prostate cancer patients on androgen deprivation therapy appeared more immature and produced more myeloperoxidase and underwent NETosis at higher rates, which may be indicative of a more immunosuppressive phenotype (97). In support of this was a study where orchiectomized mice had impaired neutrophil function and a higher burden of metastatic melanoma that was reversed with administration of testosterone (97).

Estrogen also appears to regulate neutrophil counts. In a study where neutrophils counts were assessed throughout the menstrual cycle reveal that counts increased with the peaks of estrogen levels just prior to ovulation and mid-luteal phase with a delay of 1–2 days (102, 103). This is likely due in part to an inhibitory effect of estrogen and progesterone on neutrophil apoptosis (104). Estrogen treatment not only increased levels of circulating neutrophils in mice, but also increased levels of serine proteases within neutrophils (105). Estrogen appears to enhance the ability of neutrophils to extrude DNA into the extracellular space as neutrophil extracellular traps (NETs; NETosis) (106), which is a mechanism that neutrophils use to trap and destroy invading pathogens and has more recently been shown to contributes to carcinogenesis and response to therapy [reviewed in (107, 108)]. However, progesterone inhibits NET formation and likely contributes to enhanced risk of certain infections or other complications during pregnancy (109). Estrogen also appears to regulate neutrophil phenotype and infiltration during carcinogenesis. Breast cancer risk is increased within 10 years of childbirth before becoming protective (110), which may be due in part to neutrophil behavior in the mammary gland during involution. It was shown that estrogen increases neutrophil infiltration and promotes release of cytokines and chemokines that are associated with cancer growth in the involuting mammary gland (111). By implanting ERα^−^ tumors in the involuting mammary gland, neutrophil depletion diminished the protumoral effects of estrogen on tumor growth (111). However, in a follow-up study, the authors assessed peripheral neutrophil counts and found a specific reduction in circulating low density neutrophils that are associated with cancer, though these cells had increased immunosuppressive markers (112). While it is unclear why estrogen treatment resulted in a reduction in neutrophil numbers in this instance, it indicates that there may be some instances in cancer which estrogen has an inhibitory effect on circulating neutrophil populations. That said, using various mouse models of estrogen insensitive cancer cell lines, it was shown that estrogen promotes myelopoiesis, resulting in increased accumulation of immunosuppressive granulocytic and monocytic MDSCs that promoted tumor growth (113). Mechanistically, ERα signaling promoted Jak2/Stat3 and activity in bone marrow myeloid precursors that promoted granulocytic MDSC expansion, while Jak2 and Src activity were important for monocytic MDSC expansion (113).

## Mast cells

Mast cells have been shown to express AR (114), ERα (115), and PR (115, 116). Mast cell numbers do not appear to be affected by androgens, but estrogen with or without progesterone increases mast cell recruitment to the uterus and induce their maturation and degranulation (117). Similarly, mast cell numbers are influenced by estrogen levels in rat mammary glands, with higher estrogen levels associated with increased mast cell numbers (118). Estrogen rapidly instigates ERα-dependent mast cell degranulation in bone marrow-derived mast cells, but not from mast cells derived from ERα knockout mice or mice treated with ER antagonists (116). These rapid effects indicate a non-genomic mechanism of action, with ERα localized at the plasma membrane regulating its rapid effects (119).

Progesterone appears to have mixed effects on mast cell function, which may be due to tissue localization or alternatively that progesterone triggers selective release of factors. Progesterone has been shown to inhibit histamine release from mast cells when stimulated *in vitro (*120). While progesterone did not trigger histamine release, it did trigger release of serotonin from mast cells in a dose- and time-dependent manner (121). Other studies have shown that progesterone promotes mast cell recruitment to the uterus and facilitates degranulation, which may help prepare the uterus for implantation (117). The role of hormones in mast cell function can by highlighted by fluctuations in severity of certain dermatologic disorders during the menstrual cycle and pregnancy (122). For example, appearance of hives/wheals in patients with chronic spontaneous urticaria (CSU) can worsen in a subset of patients. While often idiopathic, CSU is also associated with autoimmunity in approximately 33% of cases and occurs at about twice the rate in females compared to males. Symptoms most frequently worsen prior to menstruation when progesterone levels are high, but may also fluctuate with pregnancy, menopause, and use of hormonal contraceptives or hormone replacement therapy (123–126). This phenomenon is not to be confused with estrogen or progestogen hypersensitivity, which are often associated with previous exposure to oral contraceptives (127, 128). Patients develop IgEs that recognize estrogen or progesterone and cause similar symptoms as CSU and manifest as a cyclic form chronic urticaria that arise during either during the follicular phase of the menstrual cycle when estrogen is high or the luteal phase during peak progesterone levels (127, 128).

Testosterone does not appear to regulate IgE-dependent mast cell degranulation (114). However, testosterone was shown to induce IL-33 transcription, which is important for mast cell survival, maturation, and function (129, 130). IL-33 is associated with Th2-type cytokines and has been shown to be associated with susceptibility to EAE (129), to regulate a pro-tumor microenvironment in colitis-associated colorectal cancer (131), and to promote gastric cancer by crosstalk with macrophages (132) and by stimulating Treg-mediated suppression (133), among other functions [reviewed in (134)].

## Basophils and eosinophils

While homeostatic eosinophil numbers tend to be equal between males and females, eosinophil numbers tend to be increased in females under pathologic conditions compared to males (135). Androgens appear to decrease eosinophil numbers; orchiectomy of male mice results in increased peripheral eosinophil numbers in response to *B. pahangi* infection, while administration of testosterone to female mice reduced eosinophil numbers (136). Hormones also appear to influence the ability of eosinophils to migrate into tissues. Treatment of eosinophils with estrogen increased adhesion to endothelial cells, while testosterone reduced adhesion and viability (137). Estrogen and progesterone also increase effector function by promoting eosinophil degranulation (137), though progesterone does not appear to signal via PR-A or PR-B (138). Notably, eosinophils have been shown to have an inverse correlation with cancer risk in several malignancies, including colorectal cancer (139–144), melanoma (142, 143, 145, 146), and hepatocellular carcinoma (147), and their presence is associated with good outcomes (148).

Circulating basophils do not appear to be affected by testosterone treatment (54), but do appear to be regulated by cycling hormone levels during menstruation. As described above, neutrophil levels peak coinciding with the two spikes of estrogen during the menstrual cycle (102). However basophils circulated at inverse levels with troughs that coincided with neutrophil peaks, indicating an inhibitory effect of estrogen on circulating levels of basophils (102). However, estrogen appears to enhance histamine release from basophils stimulated with IgE (149), while progesterone has been shown to inhibit histamine release from basophils in patients with urticaria (150). Basophil counts are associated with favorable outcomes in melanoma, ovarian cancer, colorectal cancer, NSCLC, and glioblastoma, but worsened outcomes in prostate, bladder, and gastric cancers (151).

## NK cells

Males tend to have higher numbers of NK cells compared to females that produce high levels of IFNγ after viral infection. Despite the increased number, NK cell effector function is decreased in males compared to females, in part due to *Kdm6a* (UTX) escape from X inactivation, expression of which improves effector function (152). Treatment of NK cells with estrogen resulted in a decreased cytotoxicity independent of ERα expression (153). Additionally, in the uterus, it appears that estrogen and progesterone regulate NK cell migration in part via upregulation of L-selectin and α4 integrin (154), as well as secretion of CCL2 (155). Testosterone appears in increase NK activity; NK cells had increased expression of IL-12 receptor subunits and enhanced IFNγ responses in transgender men receiving testosterone, which was directly attributed to AR signaling rather than loss of ER signaling (61).

## Sex and the immune response to therapy

The sexual dichotomous nature of the immune system is well established, but how these differences affect response to cancer therapies is at its infancy. Several meta-analyses of outcomes indicate that males may benefit from immunotherapy more than females for many cancers (156–158), though some of the data are contradictory dependent on which immunotherapies are given (159), and yet others have found no benefit (160) [for a thorough review of clinical data, see reference (51)]. Within individual cancers, there are a few notable instances in which females fare better. When combined with chemotherapy, females responded better to immunotherapy than males in NSCLC (161). In MSI-high colorectal cancer, additional BRAF mutations led to better immunotherapy outcomes in females, compared to males that was not observed in BRAF wt cohorts (162). In the BRAF-mutated MSI-high cohort of males, there was a signature associated with androgen receptor signaling and an immune depleted phenotype (162). Androgen receptor activity was recently shown to be associated with poor response to immune checkpoint blockade in prostate cancer (163). Mechanistically, AR directly binds to the *Ifng* promoter and its blockade resulted in increased IFNγ expression and cytokine production in CD8^+^ T cells when combined with anti-PD-1 (163). AR blockade has also been shown to increase MHC-I expression on prostate cancer cells and improve T cell responses and tumor control (164). Similarly, AR was shown to be associated with T cell dysfunction and response to PD-1/PD-L1 blockade in colorectal cancer (165) and bladder cancer (166). High AR gene expression signatures are associated with poor responses to immune checkpoint blockade in melanoma, NSCLC and mixed tumor cohorts (167)and poor responses to BRAF/MEK-targeted therapy in melanoma (168).

In females, estrogen has been shown to promote tumorigenesis of many cancers, including ER^-^ cancers due to its immunosuppressive effects in macrophages and other cells within the TME (53, 169). Anti-estrogen therapies have shown promise in preclinical studies, and it was shown that the anti-estrogen fulvestrant improved lung cancer cell responses to immune- and chemotherapy-mediated cytotoxicity both *in vitro* and *in vivo (*170). Supporting these findings is a study where estrogen receptor signaling promoted myelosuppression in a mouse model of melanoma (171). In this study, ER signaling in macrophages promoted an M2-like phenotype in macrophages and supported tumor growth. They confirmed in clinical samples that genes associated with estrogen signaling in macrophages predicted poor response to anti-PD-1 therapy in melanoma. Loss of ERα or treatment with fulvestrant reprogrammed macrophages to an M1-like phenotype, and improved adaptive immune control when combined with immune checkpoint blockade in mice (171). However, the effects of estrogen may differ between sexes or may be context-dependent. In obese male mice, increased presence of adipose-associated estrogen was associated with increased melanoma growth, but paradoxically improved response to PD-1 checkpoint blockade (172). Here, estrogen was acting on DCs and improved cross presentation of tumor antigens to CD8^+^ T cells. This phenomenon was also observed in humans, where increased estrogen levels were associated with improved response to PD-1 blockade in obese males (172).

Presence of certain myeloid cells are associated with improved outcomes to immune checkpoint blockade. It was recently shown that IFN-stimulated Ly6E^hi^ neutrophils are a predictor of positive responses to PD-1 blockade in multiple tumor types that included urothelial bladder cancer, glioblastoma, NSCLC, renal cell carcinoma, melanoma, and stomach adenocarcinoma (173). The IFN-induced neutrophils exhibited activation of the STING pathway, which promotes type I IFN production and led to improved responses in mice (173). Similarly, increased eosinophil numbers have been associated with positive outcomes in patients treated with anti-CTLA4 and/or anti-PD-1 blockade in melanoma (174–176), NSCLC (177, 178), renal cell carcinoma (179), and triple negative breast cancer (TNBC) (180). In TNBC, increased eosinophil-related signature positively correlated with a CD8^+^ T cell inflamed signature, an IFNγ signature, and a structural CD8^+^ T cell signature in responders much more strongly than non-responders (180). Mechanistically, preclinical modeling demonstrated that improved outcomes were dependent on CD8^+^ T cells, eosinophils, and CD4^+^ T cell release of IL-5 (180). Basophil presence has also been associated with positive responses to immune checkpoint blockade in melanoma (181) and NSCLC (182), but negative outcomes in gastric cancer (183). While presence of these cells is associated with improved outcomes in various cancers and are influenced by sex hormones, it remains to be determined to what extent sex or hormones additionally influence presence or effector function of these cell types and regulate therapeutic responses.

Conversely, macrophage and MDSC presence is associated with worsened outcomes across multiple tumor types. Macrophages have been shown to contribute to all stages of carcinogenesis and impact therapeutic responses [extensively reviewed in (184–190)]. Their presence within tumors has been associated with poor outcomes across virtually all tumor types (191, 192). Given their roles in promoting carcinogenesis, they have become an attractive target for therapeutic intervention. Macrophages exist in tumors across a wide range of phenotypes rather than the binary M1/M2 historical phenotypes, and their plasticity across phenotypes adds to the complexity of identifying combination therapies (193). Of note, macrophage depleting strategies have been largely disappointing in the clinic, potentially due in part to enhanced recruitment of MDSCs (194), which are also associated with poor outcomes (195). Across these cell types, there are a wide array of targets entering clinical evaluation. Given the heterogeneity of these cell types and the number of mechanisms by which they can confer immunotherapeutic resistance, it is likely that patient stratification will need to occur for many of these targets to be effective.

Many of sex- and hormone-specific effects outlined above in immune cells should remain in effect in the context of cancer therapy. However, there are likely caveats that may be due to mutation- or TME/tissue-specific effects that impart differences in hormonal effects on the immune system. As noted above, neutrophils are increased in response to estrogen under many circumstances but were paradoxically reduced by estrogen in one study (112). Similarly, BRAF mutations in MSI-high colorectal cancer switched an otherwise male-dominated immunotherapy response into a female-dominated response (162). Additionally, there are tumor microenvironmental effects that drive imbalances in the sensing of adjuvants that drive immune responses. Radiation therapy and chemotherapies have the capacity to drive immunogenic cell death, where adjuvants released by dying cells are sensed by immune cells, including TLRs (196). As described above, sex and hormones affect TLR expression and may impact response to therapy, and there are some reports of females having better responses when radiation therapy is given alone or combined with chemotherapy in NSCLC (197, 198), brain metastasis (199), and esophageal squamous cell carcinoma (200). Interestingly, females also showed enhanced abscopal responses when radiation therapy was combined with immune checkpoint blockade across metastatic solid tumors (201). While part of the explanation might be through enhanced TLR/TRIF signaling and type I IFN production, another potential mechanism where radiation therapy may have sex-dependent effects is through activation of the cGAS/STING pathway. Radiation therapy induces DNA damage that gets released to the cytoplasm. Double-stranded DNA is recognized by GMP-AMP synthase (cGAS) which catalyzes the transformation of ATP and GTP into the secondary messenger 2’,3’-cGAMP. Stimulator of interferon genes (STING) becomes activated and promotes production of type I IFN through the TBK-IRF3 pathway. While there is scant evidence of a female bias in the cGAS-STING pathway within tumors responding to radiation therapy, there is evidence that enhanced cGAS-STING activation in female mice contributes to the female-biased vulnerability to Alzheimer’s disease (202) and in some female reproductive diseases. Together the enhanced type I IFN can promote DC maturation and cross presentation to CD8^+^ T cells. However, it remains to be determined whether the enhanced response to radiation in females in some cancers is due to these factors. While these effects demonstrate that radiation therapy can promote anti-tumor immune responses, some TLRs also signal through the MyD88/NFkB pathway to drive inflammatory gene expression. Macrophages within tumors have been shown to overexpress NFκB p50, and its expression preferentially drives immunosuppression and poor prognosis (203). Thus, when TLR ligands are sensed in the context of radiation and chemotherapies, this may preferentially drive an immunosuppressive response rather than immunostimulatory response in some instances due to TLR/MyD88/NFκB signaling by myeloid cells (204). There are likely many other sex- and hormone-based differences that may be exploited by evolving tumors to escape immune recognition and thwart cytotoxic responses and a deeper understanding of how hormones regulate immunosuppression is critical to developing rational combinatorial strategies to improve outcomes across cancer types.

## Conclusions

Sex, hormones, and the immune system are inextricably linked. The immune system is quite different between males and females, and we are only beginning to understand how sex and hormones impact the immune system during carcinogenesis and cancer therapy (51). Further complicating sex differences is that there seems to be abundant tissue specificity in these differences. In an assessment of 44 different tissues, 18.2% of all sex-biased gene expression differences were only expressed in a single tissue (205). While the vast majority of the tissues examined were not immune cells, this phenomenon of sex- and tissue-distinct differences appears to hold in macrophages from different anatomical sites (50). Thus, much remains to be understood about different immune cells in different cancer types and how their response affects therapeutic responses in males and females.

Studies in rodents have greatly informed our understanding of the human immune system, but there are important distinctions between human and mouse, and between different strains of mice. Laboratory mice generally lack the genetic diversity found in humans and are not exposed to a variety of pathogens, thereby limiting the ability of mice to recapitulate the human immune experience (206). Additionally, there are a number of genes found in human immune cells that are not expressed in mice that may mediate novel immune functions (207). Aside from these characteristics, there are also distinctions between hormone biology between mice and humans. Female mice have a 4-day reproductive cycle while humans have a 24-34-day cycle, and human females experience menopause while rodents do not (208). Additionally, humans express sex hormone binding globulin (SHBG), that binds to testosterone, DHT, and estrogen and helps control their bioavailability in tissues (209), while rodents do not express this protein postnatally (210). There are also differences in the hormonal output of the adrenal gland, where dehydroepiandrosterone (DHEA) and androstenedione are not produced in mice because they lack the enzyme 17,20-lyase in adrenal glands (211). There are several strategies being employed to address some of the shortcomings of mouse models, including utilizing humanized mice (212) or dirty mice (206, 213), or altering the hormonal milieu for more accurate xenograft studies (214). However, the above factors and other factors not discussed here may contribute to some distinct responses between sexes and immune subsets between mouse and humans, and it remains important to confirm findings in human models when possible.

Immunotherapy has changed the way cancer is treated, particularly amongst advanced cancers. While many patients benefit, only a minority of patients achieve durable, long-term responses in most cancer types. There are many studies that identify immunosuppressive myeloid cells as a barrier to treatment responses and efforts are underway to modulate these responses in combination with chemotherapy, radiation therapy, and checkpoint inhibition to improve responses in a greater number of patients (215–218). There may be opportunities to target these cells in a hormone and sex-biased manner, but more needs to be done to understand these responses by reporting sex- and hormone-stratified results. Additionally, personalized and off-the-shelf vaccines are being tested in the clinic with varying degrees of success (219, 220). Given the roles of hormones in regulating DC response, it is plausible that vaccine timing during the menstrual cycle may influence efficacy for younger patients. While data are scarce, it was recently shown that COVID-19 vaccination in females during the follicular phase had a 35% increased likelihood of self-reported side effects, and while underpowered due to few breakthrough infections, there was a 5-week longer median time to infection than those vaccinated in the luteal phase (221). Additional studies need to be done to understand how sex and hormones impact in cancer vaccine responses in trials, where vaccine immunogenicity may be highly variable.

As humans age, cancer risk increases and hormone levels drop, likely impacting the magnitude of the hormonal effects of hormones between male and female immune systems. However, immune function also changes with age due to changes in immune cell numbers and epigenetic changes that alters the balance between adaptive and innate immunity. In aged individuals, the innate immune system is detrimentally affected. The total macrophage population increases as a function of age, yet have decreased functionality (222–224). Aged macrophages respond less effectively to TLR agonists, are less phagocytic, and become senescent, secreting more proinflammatory cytokines that impair T cell function (224–228). Interestingly, many tissue-resident macrophages that are derived from the yolk sac are replaced by bone marrow-derived macrophages during aging (229). Yolk sac-derived macrophages have been shown to expand during pancreatic carcinogenesis and contribute to cancer-associated fibrosis and progression (230, 231), but how aging shapes this response remains unclear. Neutrophils similarly increase as a function of aging, yet are less active (224, 232). They have reduced phagocytic capacity, migrate less effectively, and have reduced ROS production (233, 234). Additionally, there are notable differences in the effects of aging between males and females, despite the decrease in hormones (235). While both sexes exhibit declines in T cell function and increases in NK, memory CD8^+^ T cell, and monocyte function in PBMCs, males displayed a greater increase in monocyte activity and inflammation (235). There was also an opposing effect of aging on B cell function, where males exhibited a significant decrease in B cell function while females displayed increased B cell function (235). Additional studies are needed to determine how these age-related immune changes impact therapeutic responses.

Most cancers are diagnosed in older patients when the hormonal differences are narrower; however, incidence of certain cancers (e.g. breast, cervical, colorectal, liver, pancreas, kidney, and stomach) is relatively high or increasing in patients younger than 50 in the United States (5, 236) when higher levels of circulating hormones remain comparatively elevated. Given the link between hormone signaling and immune responses, it is imperative to better understand how sex and hormones impact the response to anti-cancer therapies. In addition to pre-clinical studies, sex-specific analyses within larger clinical trials and sex-specific clinical trials will help clarify how sex impacts the immune response to therapy within and across particular cancers. Sex stratification has become routinely reported in trials, but inclusion of menstrual cycle, menopausal status, or hormone therapy use is not routinely reported. Inclusion of these criteria would help identify the clinical impact of these variables and how they impact the immune response to chemotherapies, radiation therapy, and immunotherapies. Inclusion of these metrics would help to identify sex- and hormone-responsive therapies that can be used to stratify patients, and to improve outcomes for cancer.
